# Prostate-Specific Antigen as an Ultrasensitive Biomarker for Patients with Early Recurrent Prostate Cancer: How Low Shall We Go? A Systematic Review

**DOI:** 10.3390/biomedicines12040822

**Published:** 2024-04-08

**Authors:** Finn Edler von Eyben, Kalevi Kairemo, Daniel S. Kapp

**Affiliations:** 1 Center of Tobacco Control Research, DK-5230 Odense, Denmark; 2 Department of Molecular Radiotherapy & Nuclear Medicine, Docrates Cancer Center, FI-00185 Helsinki, Finland; kalevi.kairemo@gmail.com; 3 Department of Radiation Oncology, Stanford University School of Medicine, Stanford, CA 94305, USA

**Keywords:** prostate-specific antigen (PSA), PSA half-life, PSA nadir, PSA relapse (PSAR)-prone prostate cancer, PSA threshold, survival, systematic review, ultrasensitive PSA assays

## Abstract

Serum prostate-specific antigen (PSA) needs to be monitored with ultrasensitive PSA assays (uPSAs) for oncologists to be able to start salvage radiotherapy (SRT) while PSA is <0.5 µg/L for patients with prostate cancer (PCa) relapsing after a radical prostatectomy (RP). Our systematic review (SR) aimed to summarize uPSAs for patients with localized PCa. The SR was registered as InPLASY2023110084. We searched for studies on Google Scholar, PUBMED and reference lists of reviews and studies. We only included studies on uPSAs published in English and excluded studies of women, animals, sarcoidosis and reviews. Of the 115 included studies, 39 reported PSA assay methods and 76 reported clinical findings. Of 67,479 patients, 14,965 developed PSA recurrence (PSAR) and 2663 died. Extremely low PSA nadir and early developments of PSA separated PSAR-prone from non-PSAR-prone patients (cumulative *p* value 3.7 × 10^12^). RP patients with the lowest post-surgery PSA nadir and patients who had the lowest PSA at SRT had the fewest deaths. In conclusion, PSA for patients with localized PCa in the pre-PSAR phase of PCa is strongly associated with later PSAR and survival. A rising but still exceedingly low PSA at SRT predicts a good 5-year overall survival.

## 1. Introduction

It is debated how best to treat high-risk patients with localized prostate cancer (PCa) [1,2,3]. After the initial treatment given with curative intention, up to half the patients recur. The first phase of recurrence is a prostate-specific antigen (PSA) relapse (PSAR). Radiation oncologists use radiation therapy to treat patients with PSAR after a radical prostatectomy (RP) [4,5,6].

PSA is the main biomarker for PCa. The serum total PSA (PSA in our systematic review (SR)) is widely used in the screening, staging and monitoring of treatment. The D’Amico classification of localized PCa has three risk groups based on PSA, PCa pathology and the extent of PCa [7]. Guidelines recommend oncologists start SRT at rising PSA < 0.5 ng/mL (<500 ng/L) [8]. Until 2021, the European Association of Urology (EAU) recommended a PSA threshold > 0.2 ng/mL (>200 ng/L) to diagnose PSAR. But, recently, EAU guidelines abandoned the threshold. A short PSA doubling time (PSADT) points to patients who develop metastases for patients with nonmetastatic castration-resistant PCa (nmCRPC) and failure to androgen deprivation therapy (ADT) [9].

If PSA is measured with a conventional PSA assay, patients with localized PCa cured with radical prostatectomy (RP) are reported to have an unmeasurable PSA nadir. But ultrasensitive PSA assays (uPSAs) can measure PSA nadir for these patients [10]. These assays quantify extremely low PSA values. PSA is not specific for PCa. Cured PCa patients have measurable, but extremely low, PSA nadir because their urethra, breast and other tissues produce a little PSA [11,12,13,14]. Women also have detectable, but extremely low, PSA, despite not having a prostate [15,16,17,18].

Many RP patients with PSA nadir < 10 ng/L do not later develop rising PSA and remain free of recurrence, while RP patients with a higher PSA nadir and later a continuous rise in PSA tend to develop a diagnosed recurrent PCa. Oncologists debate whether patients with PSAR benefit from SRT being started at extremely low but rising PSA (exceedingly early SRT). Our SR aims to evaluate whether PCa-recurrent patients treated with SRT at a rising PSA of 30–50 ng/L may live longer than those starting SRT at a much higher rising PSA.

## 2. Methods

### 2.1. Search for Studies and Analyses of the Studies

**Figure 1:**
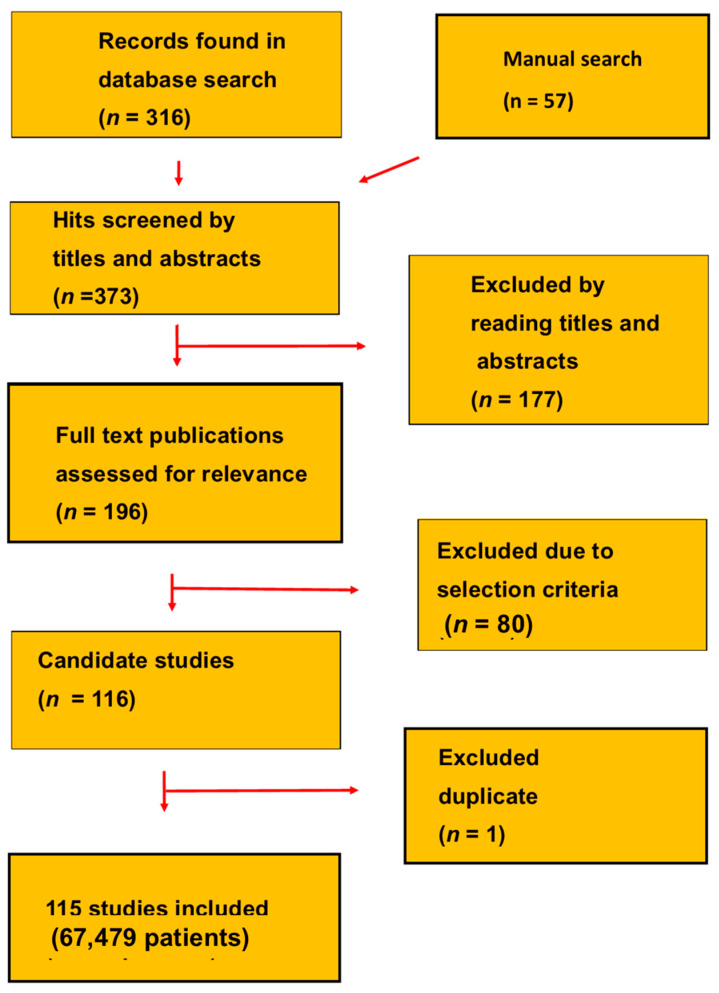
PRISMA flow scheme for the selection of studies.

Our investigation was a SR following the Preferred Reporting Items for Systematic Analysis (PRISMA) guidelines [19]. Our SR was registered as INPLASY023110084, DOI 10.377662023.11.0084. We searched on Google Scholar, PubMed and reference lists of original research studies and reviews for original research studies on total PSA measured with uPSAs. A PubMed search in May 2023 used the search words ((prostate cancer or biochemical recurrence) and (prostate-specific antigen or PSA) and (ultrasensitive or superselective or hyperselective)). The database search gave 316 hits (Figure 1 and Table 1 and Table 2). The review includes 115 studies and 67,479 patients [20,21,22,23,24,25,26,27,28,29,30,31,32,33,34,35,36,37,38,39,40,41,42,43,44,45,46,47,48,49,50,51,52,53,54,55,56,57,58,59,60,61,62,63,64,65,66,67,68,69,70,71,72,73,74,75,76,77,78,79,80,81,82,83,84,85,86,87,88,89,90,91,92,93,94,95,96,97,98,99,100,101,102,103,104,105,106,107,108,109,110,111,112,113,114,115,116,117,118,119,120,121,122,123,124,125,126,127,128,129,130,131,132,133,134].

Our SR included studies of initial-phase PCa patients that measured PSA with uPSAs irrespective of the PSA levels. Of duplicate studies, we selected those with the largest number of patients or those published most recently. Our SR excluded a study in the Japanese language, reviews, abstracts; case reports, apart from an illustrative single case report, animal studies, studies of male patients with benign prostate hyperplasia, prostatitis or metastatic castration-resistant prostate cancer (mCRPC), female patients with breast and ovarian cancer and laboratory studies of rates of free PSA to total PSA, complex PSA, proPSA, other kallikreins, and urinary PSA.

An author (FEvE) summarized the findings of the studies. Often, the studies reported the total number of patients and the percentage in the group with a specific outcome. Therefore, the author calculated the number of patients with the outcome as (the total number) times (the percentage). The result was summarized to the nearest whole number of patients. The author validated the studies in three rounds. With discordance between the rounds, our SR used the findings of the final round. The SR used Forest plots to visually display the results of the individual studies. The SR also calculated a summary *p* value for an association between characteristics based on the *p* values of the individual studies.

Most studies did not specify the generations of uPSAs used in the studies. Our SR grouped the clinical studies according to the clinical phase, either the pre-PSAR phase or nmCRPC. Our SR summarized the pre-PSA-phase studies for the association between PSA kinetics and PSAR and for the association between PSA at start of SRT and secondary PSAR.

The International Federation of Clinical Chemistry and Laboratory Medicine recommends reporting findings by the International System of Units (SI) [135]. Many countries and medical journals, like *Biomedicines*, adhere to the SI. Most studies reported the PSA values as µg/mL. Our SR followed the SI and reported PSA as µg/L and ng/L according to the SI.

### 2.2. uPSA

Specialists in laboratory medicine successively developed five generations of uPSAs. First-generation uPSAs had a low limit of detection (LLD) of 100 ng/L, and the third-generation uPSAs had a LLD of 1–10 ng/L. First-generation uPSAs had a low limit of quantitation (LoQ) of 500 to 1000 ng/L, and third-generation uPSAs had a LoQ of ≤ 10 ng/L. A fourth-generation and a fifth-generation uPSA had a LoQ of < 1 ng/L.

### 2.3. Definitions

For RP, PSAR was defined as patients who initially may have had a postoperative PSA decline to values of < 10 ng/L and later had rising PSA values without PCa lesions being identified with ultrasound, CT and bone scans. For RT patients, PSAR was defined with a Phoenix criterion for the rising PSA [136]. Our SR defined remaining PCa after the initial treatment not identified through conventional imaging as residual PCa. uPSAs were defined as assays that could quantify PSA < 10 ng/L. For PCa patients in stable remission during a long follow-up, the LoQ was defined as the lowest PSA concentration where the measured PSA varied ≤ 20% in repeat measurements.

A pre-PSAR phase of PCa was defined as the time from the initial treatment to the time where the patients fulfilled the earlier EAU criterion for PSAR, i.e., 0.2 ng/mL (200 ng/L). After the treatment, the PSA half-life was defined as the time to a 50% PSA decline. PSA nadir after the initial treatment was defined as the lowest PSA in series of PSA measurements. The PSA doubling time (PSADT) was defined as the time for PSA to double its value. After RP, PSAR-prone patients were defined as those who after the post-operative PSA nadir (often < 50 ng/L) had a continuous rise in PSA. After RP, non-PSAR-prone patients were defined as those who after the post-operative PSA nadir did not have a continuous rise in PSA.

The performance of a PSA assay was defined by the diagnostic specificity, diagnostic accuracy, predictive value of a positive test and predictive value of a negative test. Bias was defined as the deviation between the measured PSA and the real PSA in the samples based on the WHO’s PSA reference standards. Precision was defined as the analytic and biologic variation in PSA measurements.

For RP patients, exceedingly early SRT was defined as SRT of recurrent patients with rising PSA < 50 ng/L. Early SRT was defined as SRT of recurrent patients with rising PSA of 200–500 ng/L at the start of treatment [137]. Late SRT was defined as SRT of recurrent patients with PSA > 500 ng/L. Recurrent patients who were given SRT at a rising PSA of 50–199 ng/L were defined as the intermediate group of patients.

### 2.4. Statistical Analysis

Our SR evaluated the diagnostic performance of uPSAs in two-by-two tables of PSAR-prone and non-PSAR-prone patients against patients with PSA above or below a PSA threshold. We calculated Forest plots of binomial data according to a STATA program developed by Nyaga et al. [138]. We analyzed whether PSA nadir after the initial treatment was associated with recurrence and overall survival (OS). We calculated an overall *p* value for the impact on an outcome using the *p* values reported in relevant studies, using the method developed by Tobias [139]. Linear regression analyses evaluated whether two clinical characteristics were significantly associated. Our SR considered a *p* value < 0.05 as statistically significant. We carried out the statistical analyses using STATA version 16.0 with updates (Stata Corp., Station, TX, USA).

## 3. Results

### 3.1. Selected Studies

Our SR included 115 studies (Supplementary Table S1). A total of 36 studies reported uPSA assays and 79 reported clinical findings. The studies were published between 1992 and spring of 2023. The studies included 67,479 patients with a median 300 patients (IQR 148–754 patients) in the individual studies. Four studies reported >2000 patients. For PSAR patients median age at diagnosis of PCa was 64 years and for patients with high-risk castration-resistant nonmetastatic PCa (nmCRPC), the median age was 74 years. Follow-up varied from 2.5 to 13 years. In the studies after the initial treatment, 14,965 patients developed PSAR and 2663 died.

### 3.2. uPSAs

The 36 studies of uPSAs were summarized in Table 1. Specialists developed five generations of uPSA assays. Five uPSA assays had registered trade-marks. Mione et al. reported a third-generation uPSA assay [126]. Wilson et al. reported a fifth-generation uPSA assay [90], and so did Ren et al. (Mesoscale) [41] and Sokoll et al. (AccuPSA) [70]. Hahn et al. reported a colorimetric switchable linker-based assay, where the LLD (100 ng/L) was more sensitive than the LLD of a conventional ELISA assay (23 µg/L) [46]. Heydari-Bafrooei et al. reported an electrochemical assay [57]; Kavosi et al. reported an electrochemical immunosensors assay [70]; and Lepor et al. reported another PSA assay [85]. Mani et al. reported a gold nanoparticle and multienzyme-particle amplification assay [96], and Morris et al. reported a Bayer immune 1 PSA assay [118].

Soletormos et al. studied the variation in PSA measured with conventional PSA assays [140]. The assays had a high imprecision.

Our SR summarized efforts to standardize PSA assays. uPSA assays differed in diagnostic performance. A study compared four uPSA assays [141] and a second study compared six uPSA assays [142]. Standardized assay methods reduced the variability in PSA measurements. In 1999, the WHO created a WHO reference preparation 96/670. Later, it was renewed in a WHO preparation 17/100 [143]. Further studies showed uPSA assays had a good accuracy and precision towards the WHO preparation [144,145,146,147,148].

### 3.3. Pre-PSAR Phase

**Figure 2:**
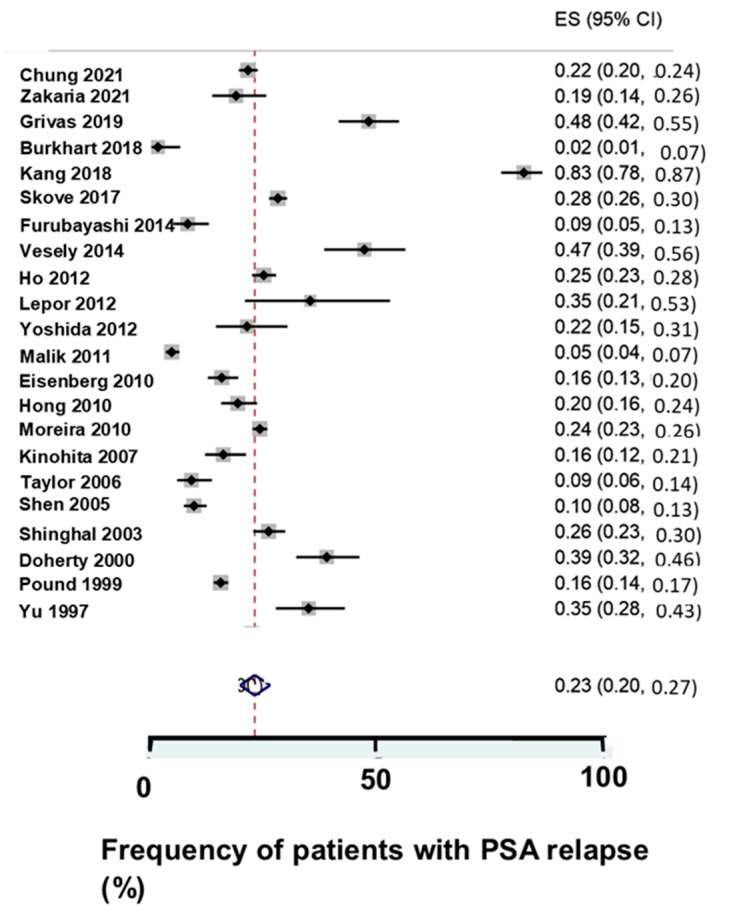
Frequency of patients with PSA relapse (PSAR) in studies of PSA nadir following radical prostatectomy (RP). The studies varied in frequency of PSAR after RP following radical prostatectomy [22,34,36,45,52,53,60,77,81,84,85,88,89,93,94,101,107,109,110,111,135,136].

**Figure 3:**
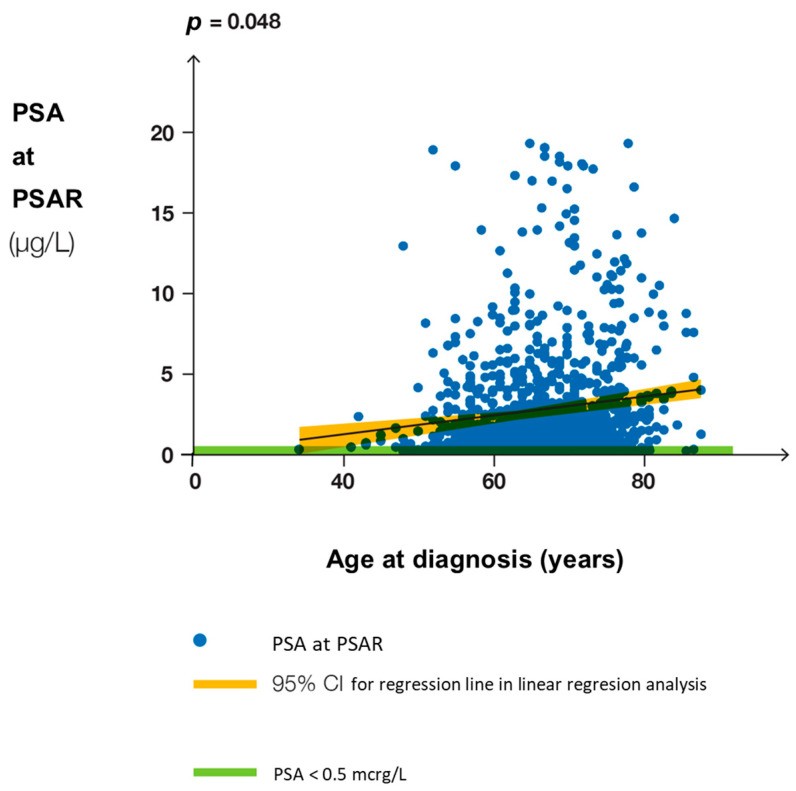
Scatterplot of pretest PSA in relation to age at diagnosis based on the multicenter study of patients with PSAR by von Eyben et al. [33]. The figure was truncated at PSA 20 µg/L. Most PSAR patients were restaged at PSA value > 0.5 µg/L, the upper limit for most PSAR patients that was recommended in recent guidelines and shown in the figure with green color. PSA had a weak but statistically significant linear association with age at diagnosis.

The 76 clinical studies of PCa-recurrent patients are summarized in Table 2. In the studies, a fifth of the patients had PSAR after the initial treatment with a curative intention (Figure 2), but the studies varied in PSAR frequency. In the studies, 14,965 patients had PSAR, and 2663 patients died. A multicenter study of 1216 patients showed that PSA varied considerably, as shown in Figure 3.

A multicenter study of 1216 patients with PSAR showed that PSA at restaging varied considerably as shown in Figure 3. Most patients had a restaging PSA of >0.5 µg/L (>500 ng/L), with patients < 50 years having a lower restaging PSA than older patients. The restaging PSA correlated with age at diagnosis, but an increasing age was only modestly associated with a rise in PSA.

**Figure 4:**
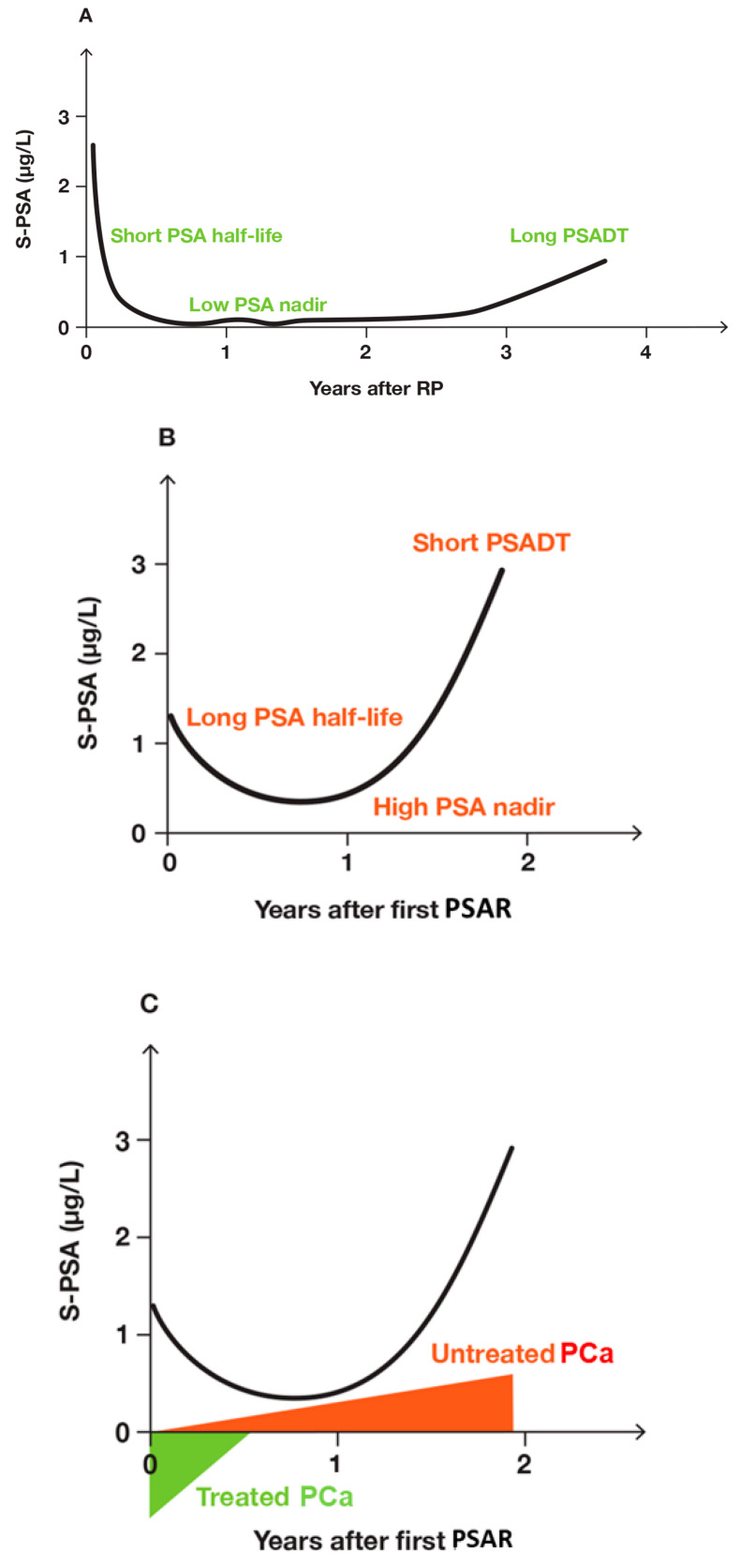
Changes in PSA for a patient who initially had aggressive PCa with bilateral lymph node metastases. (**A**) Clinical course up to first PSAR. (**B**) Clinical course up to second BCR. (**C**) Explanatory model indicating how changes in measured PSA (S-PSA) reflected PSA released from treated PCa lesion (green) and untreated PCa lesions (red) up to the second PSAR.

**Figure 5:**
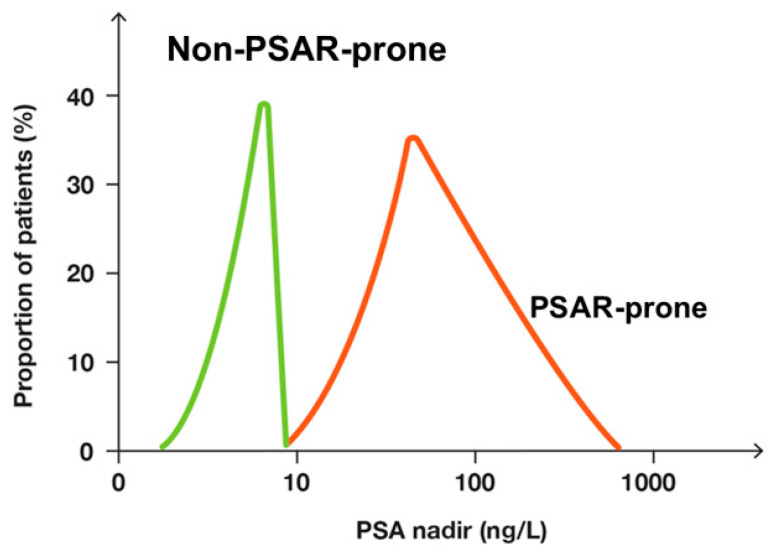
Distribution of PSA nadir after radical prostatectomy for PSAR-prone patients (red curve) and non-PSAR-prone patients (green curve). The x-axis shows PSA values on a logarithmic scale.

For a patient who underwent RP, as an example, PSA changed after RP to a first and a second PSAR (Figure 4) [149,150]. After the first PSA nadir, PSA rose slowly (Figure 4A), until a restaging [^18^F]FACBC PET/CT showed the patient had a lesion in a pararectal lymph node. It was treated with SRT. Later, the patient had a second PSAR (Figure 4B). At the second PSAR, the tumor burden was higher than it had been at the first PSAR. The larger tumor burden prolonged the PSA half-life and increased the second PSA nadir. However, following RP, the time to the first PSA nadir (TTN) was 8 months, and so was the TTN to the second PSA nadir (Figure 5).

Before the second PSA nadir, the measured PSA included both residual PSA derived from the irradiated PCa lesion, new PSA from growing PCa lesions not included in the field of the radiation therapy and PSA from other organs, as explained in Figure 4C.

After RP, the PSA nadir is important. Kang et al. reported that more patients with a PSA ≥ 0.3 µg/L (≥300 ng/L) three months after RP developed PSAR than patients with a PSA < 0.3 µg/L (<300 ng/L) [53]. Chung et al. reported that non-PSAR-prone patients had a lower PSA nadir than PSAR-prone patients [34]. Grivas et al. reported that after RP, PSA nadir ≥ 0.2 µg/L (>200 ng/L) significantly predicted PSAR [45]. Lepor et al. reported PSAR-prone and non-PSAR-prone patients differed markedly in PSA nadir [85], as shown in Figure 4. Skove et al. reported that the PSA nadir after RP had an impact on development to PSAR [60]. More patients with a detectable PSA developed PSAR than patients with an “undetectable” PSA, and the ten-year PSAR-free survival was 70% vs. 30%, respectively. Lepor et al. reported that PSAR-prone and non-PSAR-prone patients differed in PSA nadir [85]. Sokol et al. reported that RP patients with a PSA nadir after RP < 0.01 µg/L (<10 ng/L) lived longer than the patients with a PSA nadir > 0.01 µg/L (>10 ng/L) [67]. Kinoshita et al. reported that PSA nadirs were significant for outcome [101].

PSA at initiation of SRT is important for the survival of the patients. Lee et al. reported that patients with a PSA ≤ 0.5 µg/L (≤500 ng/L) at the start of SRT lived longer free of new metastases than patients with a PSA > 0.5 µg/L (>500 ng/L) [22]. Tilki et al. reported that more patients with a PSA > 0.25 µg/L (>250 ng/L) at the start of SRT died during the follow-up than patients with a PSA ≤ 0.25 µg/L (≤250 ng/L) [25].

Bottke et al. reported of patients with PSAR where more patients with a PSA > 0.2 µg/L (>200 ng/L) at the start of SRT later progressed than patients with a PSA < 0.2 µg/L (<200 ng/L) [43]. Further, SRT reduced PSA to “unmeasurable” PSA values for 91% of the patients with PSA at SRT < 0.2 µg/L (<200 ng/L). Multivariate analyses showed that nadir PSA after SRT was more highly significant for the prognosis than PSA at SRT. Stish et al. reported that PSAR patients with a PSA < 0.5 µg/L (<500 ng/L) at the start of SRT lived longer without a second relapse than patients with a higher PSA at SRT [68].

In the study by Kinoshita et al. only the PSA nadir had a *p* value < 0.0001 for the prediction of outcome [101]. In comparison, the preoperative PSA, clinical stage, and Gleason score had higher but still significant *p* values: 0.001 to 0.04.

Von Eyben et al. reported that PSAR patients with a pretest PSA < 0.5 µg/L (<500 ng/L) lived longer than patients with a pretest PSA > 0.5 µg/L (>500 ng/L) [32]. Dess et al. analyzed the RTOG 9501 prospective randomized trial (RCT) that evaluated RT of the prostate bed with or without ADT [39]. Patients with PSA ≤ 1.5 µg/L (≤1500 ng/L) at RT lived much longer than those with PSA > 1.5 µg/L (>1500 ng/L) at RT.

**Figure 6:**
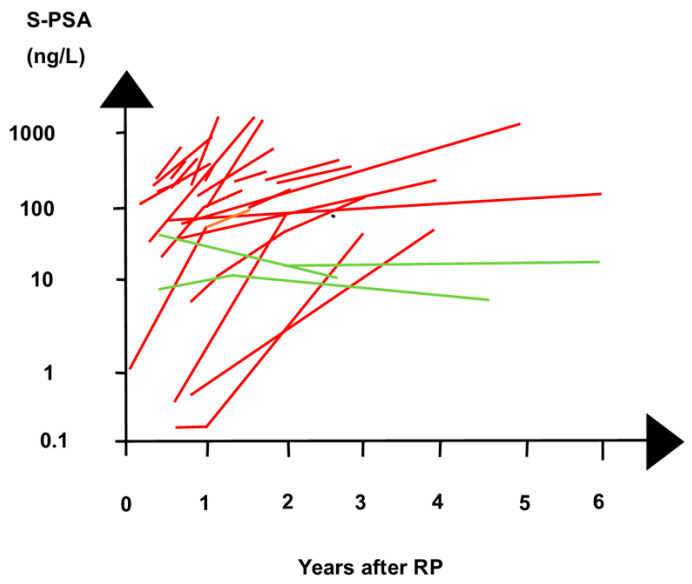
PSA relapse (PSAR) during the follow-up after radical prostatectomy (RP) (time 0). The figure combines the clinical course of PSA after radical prostatectomy (RP) for individual patients as they were shown in previous studies. The PSA values on the y-axis are shown on a logarithmic scale. Three non-PSAR-prone patients (green lines) and twenty PSAR-prone patients (red lines) differed regarding PSA kinetics following RP. Non-PSAR -prone patients had fluctuating PSA values < 30 ng/L following RP. After PSA nadir following RP, PSA increased to >50 ng/L for the twenty PSAR-prone patients before they were given salvage treatment for PSAR. Already during the first year after RP, PSAR-prone patients had rising PSA values in contrast to the falling PSA values for the non-PSAR-prone patients. The PSA kinetics separated PSAR-prone and non-PSAR-prone patients better than the numeric PSA values. The figure illustrates that within the first two years after RP, some PSAR-prone patients had an obvious rise in PSA, but were nevertheless followed-up with up to 6 years before they were started on salvage treatment. Abbreviations: S-PSA—serum prostate-specific antigen.

**Figure 7:**
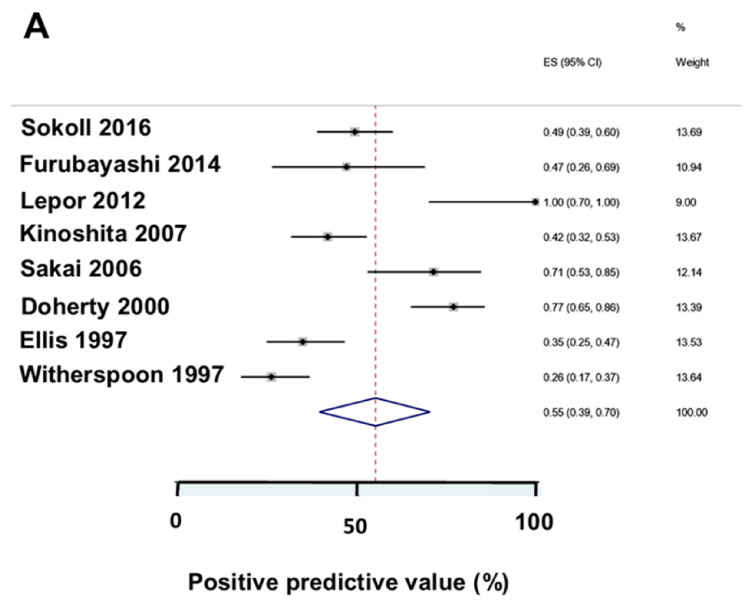

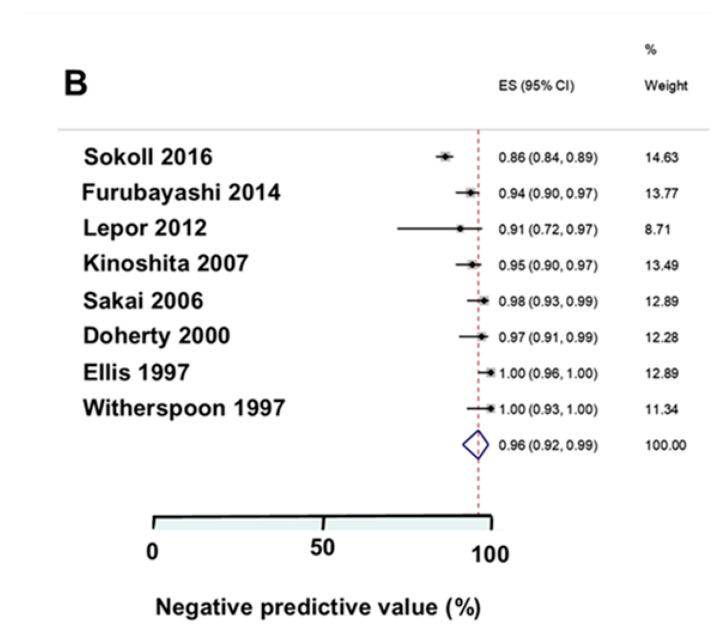
Diagnostic performance of ultrasensitive PSA assays in clinical studies of PSA nadirs after radical prostatectomy [67,77,85,101,105,111,119,121]. (**A**) Predictive values of a positive PSA test. (**B**) Predictive value of a negative PSA test. The high predictive value of a negative PSA test supported the clinical use of PSA measured with ultrasensitive PSA assays for PCa patients in the pre-PSAR phase.

Figure 6 shows the PSA nadir after RP for the patients who later developed or did not develop PSAR. Figure 7 shows that patients with or without later PSAR differed in the development of PSA.

### 3.4. High-Risk Nonmetastatic Castration-Resistant Prostate Cancer

**Figure 8:**
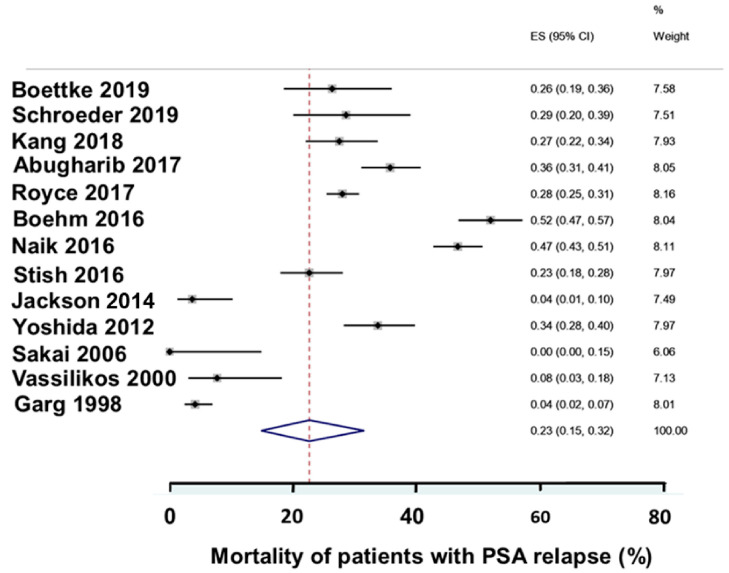
Overall mortality in studies that reported the mortality [43,49,53,56,59,63,66,68,78,88,105,112,117]. The PSAR patients varied in mortality between the studies. The mortality increased with the duration of the follow-up; therefore, differences in duration of the follow-up may have contributed to the differences in mortality.

The PROSPER RCT investigated adding enzalutamide to ADT for patients with high-risk nonmetastatic castration-resistant prostate cancer (nmCRPC). In an analysis of the RCT, Hussain et al. reported that a reduction of PSA with a PSA nadir < 0.2 µg/L (<200 ng/L) gave a better survival free of new metastases than a less extensive reduction of PSA [21]. Both patients of intermediate and poor risk had a marked impact on the OS. As reported by Saad et al. the SPARTAN RCT investigated the effect of adding apalutamide to ADT [30] (Figure 8).

**Figure 9:**
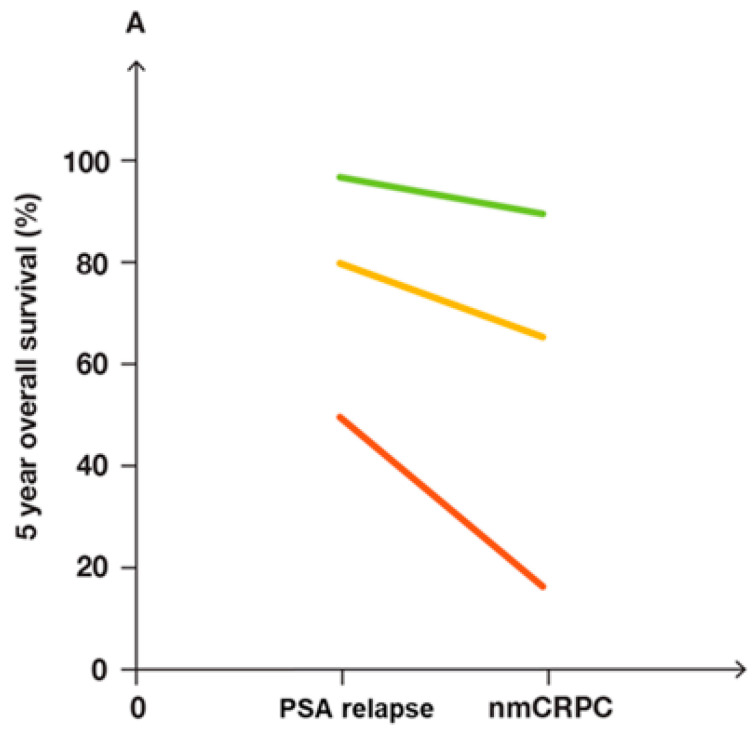

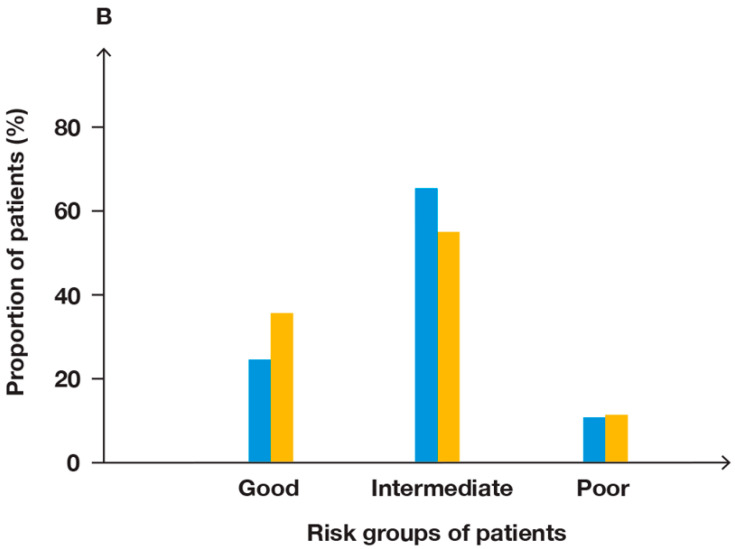
We based the five-year overall survival (OS) of the patients with PSA relapse (PSAR) on a previous study of patients restaged with PSMA PET/CT [85]. We based the OS for patients with nonmetastatic castration-resistant PCa (nmCRPC) in publications of randomized controlled trials. The three risk groups of PSAR patients and nmCRPC patients differed in five-year OS. (**A**) In the two phases of recurrent PCa, OS differed more for the subgroup of intermediate patients (yellow line) and high-risk subgroup of patients (red line) than for the low-risk subgroup of patients (green line). (**B**) PSAR patients (blue columns) and nmCRPC patients (orange columns) had similar proportions of patients in the three risk groups.

The three risk groups of patients with PSAR and nmCRPC differed markedly in the five-year OS, as shown in Figure 9.

## 4. Discussion

Our SR reported two phases of recurrent PCa based on restaging PSA after the initial treatment and illustrated that even exceptionally low PSA values related to the outcome. PSAR is an earlier phase of recurrent PCa than nmCRPC. Of the patients in the intermediate and high-risk groups, PSAR patients lived longer than the high-risk nmCRPC patients.

The D’Amico classification has two PSA thresholds (10 and 20 ng/mL (10,000 and 20,000 ng/L)) to separate patients into three risk groups [7]. The PSA thresholds were higher than our PSA threshold (30–50 ng/L). Also, the Prostate Cancer Trial Working Group version 3 (PGWG3) had a higher PSA threshold [151]. PCWG3 advise oncologists not to diagnose PSAR before a rising PSA reaches a PSA threshold of 0.2 ng/mL (200 ng/L). Further the threshold in the EAU guidelines recommended before 2021 for the diagnosis of PSAR was higher than our PSA threshold.

For RP patients, it may take up to eight weeks before PSA reduced to a post-operative PSA nadir. In the pre-PSAR phase of PCa, we called a high-risk group of patients for PSAR-prone patients. If these patients were followed-up without an active treatment, they later fulfilled the previous EAU criterion for PSAR, mainly a rising PSA up to 200–500 ng/L. An extremely low but divergent PSA could separate PSAR-prone and non-PSA-prone patient as early as three months after RP, and the difference between the two groups of patients became increasingly larger and more significant as the follow-up after RP became longer.

Many RP patients with PSA nadir < 10 ng/L have a minimal risk of recurrence [123]. Measured with uPSAs, PSA detected the recurrence of PCa earlier than if PSAR was diagnosed according to the previous EAU criterion. If SRT was started at extremely low but rising PSA values, the PCa-recurrent patients had a lead time of up to one year compared with early SRT, started at a rising PSA of 200–500 ng/L.

PSAR-prone patients had a progressive rise in PSA after the postoperative PSA nadir following RP. Patients with PSAR treated with SRT while PSA was <280 ng/L lived longer than those treated with SRT at a higher PSA [25,87]. A meta-analysis of patients treated with SRT showed the risk of a second recurrence increased 2.5% per 100 ng/L PSA rise at SRT [152]. Unfortunately, most recent PSAR patients had rising PSA of >500 ng/L at the time the salvage treatment was started, as shown in Figure 3.

A study reported that a restaging PSMA PET/CT of patients with PSAR according to the Phoenix criterion after initial RT often detected metastatic PCa. The authors indicated that for patients treated with RT in the early phase of PSAR, the Phoenix criterion needed to be updated to adequately diagnose PSAR relative to the Phoenix criterion.

PSA detected recurrent PCa earlier than restaging imaging. The multicenter study of recurrent PCa patients and other studies indicated that at PSA of 20–50 ng/L, only 20% of the patients would have had a positive PSMA PET/CT [32,33,153]. Surveys among German oncologists reported that PSAR patients with low PSA had a similar 20% frequency of positive PSMA PET/CT [154,155]. A German study of PSAR patients with a restaging PSA < 0.2 ng/mL (<200 ng/L) reported a 25% frequency in positive PSMA PET/CT [156].

Only a few patients had positive findings with multiparametric MRI when the patients were restaged at rising PSA < 300 ng/L [157].

Our SR used the criterion for PSAR EAU employed before 2021. We recommend that recurrent PCa patients are treated with SRT while a rising PSA is extremely low. Also, Diamandis et al. argued that recurrent PCa patients had an advantage if SRT was started at extremely low but rising PSA values [158].

Oncologists debate the timing of RT for patients with high-risk PCa initially treated with RP [155,159]. An argument favoring adjuvant radiation therapy (ART) is that ART leads to fewer PCa deaths than SRT [160]. A con for ART is that ART implies an unnecessary treatment for a third of the high-risk patients. An argument favoring SRT is the certainty that all RP and PSAR patients need SRT, and a con for SRT is the delay in treatment relative to ART. A German survey 2021 of restaging PSMA PET/CT reported that a tenth of the specialists did not use a PSA threshold to diagnose PSAR, a third used a PSA threshold of >0.2 ng/mL (>200 ng/L) and two thirds of the specialists used a PSA threshold of >0.5 µg/mL (>500 ng/L) [155]. Nevertheless, SRT given at extremely low PSA values combined the advantages of ART and SRT and avoided the cons.

A recent individual patient meta-analysis of three RCTs of ART and SRT showed that the treatments differed little in the five-year survival free of PSAR [5,6,161]. But, the authors of the RCTs were concerned with the participants developing recurrence less often than expected. The patients had a frequency of recurrence that was half of that in our studies. Recent studies of PSAR showed that more than half of the patients had restaging PSA above the PSA threshold of 0.5 µg/mL (500 ng/L) used in the RCTs. The consensus was that the RCTs supported SRT.

Previously, von Eyben, 2020, proposed a prospective RCT comparing patients monitored for PSA with an uPSA or monitored with a conventional PSA assay [162]. But recurrent EAU guidelines support early SRT [7]. Therefore, today, the trial is obsolete.

The 2023 National Cancer Consensus Network (NCCN) guidelines for PCa include a subgroup of patients with PSAR where PSA is measurable due to PSA from normal tissue. But, non-PSAR-prone patients have measurable PSA nadir. The non-PSAR-prone patients have an exceptionally low risk of recurrence.

A new retrospective trial is initiated to re-evaluate whether patients with PSAR after initial RP have an excellent OS if radiation oncologists start SRT while the rising PSA is <200 ng/L.

Our SR had strengths and limitations. As strengths, our SR was comprehensive and up-to-date. Our SR supported the assumption that a change for PSAR patients from early SRT to exceedingly early SRT may improve the outcome for PCa-recurrent patients, as well as any innovation of systemic treatments. As limitations, our SR did not evaluate PSA as the screening or staging of PCa. No RCT evaluated exceedingly early SRT. In our SR, only one reviewer undertook analyses of the findings of the studies, but obtained three separate evaluations of the findings.

The studies in our SR varied considerable in duration of the follow-up, and most studies had a limited follow-up of < 10 years. The heterogeneity in the duration of follow-up may explain part of the differences in mortality between the studies. A histologic type of PCa did not produce PSA [163,164]; therefore, PSA could not detect recurrence in patients with that histology. Finally, our SR only evaluated blood tests and did not evaluate PSA derivatives, circulating tumor cells [165], SCHLAP1 [166,167], other long non-coding RNA [168] or genomic classifiers [169,170].

In addition to our SR, a recent individual patient data (IPD) analysis of 10,415 patients with localized PCa treated with radiation therapy with or without ADT reported a consistent prognostic value of a PSA threshold of 0.10 ng/mL (100 ng/L) for the PSA nadir up to six months after the initial radiation therapy [171]. The patients had a median PSA at randomization of 13.2 ng/mL (IQR 8–24). The PSA threshold during the first six months of follow-up significantly predicted the outcome.

## 5. Conclusions

The clinical use of exceedingly low but rising PSA values was shown to be promising for patients with localized high-risk PCa who may develop recurrence after the initial treatment.

## 6. Perspectives

An estimated 1.5 million patients worldwide develop PCa each year, an estimated 400,000 patients developed PSAR and an estimated 70,000 patients die each year. A shift from the conventional measuring of PSA to measuring PSA with a third-generation uPSA and reporting PSA as ng/L could detect recurrent PCa exceedingly early and assist in the clinical management. The shift is a challenge. In the pre-PSAR-phase, a measurable PSA may not indicate PSAR, but for many patients in this phase, the shift from having “unmeasurable” PSA to having extremely low but rising PSA may have clinical implications.

The improved implementation of the present goal for early salvage treatment implies a consensus between oncologists. A consensus is especially needed for exceedingly early SRT.

Our SR gave a perspective for patients with high-risk PCa. In patients with recurrent PCa, the detection of early but rising PSA values facilitated exceedingly early SRT. A shift from early SRT to exceedingly early SRT did not change the adverse effects or the costs of the SRT. Compared with early SRT, exceedingly early SRT may reduce the risk of later metastatic PCa and death as a result of PCa.

## Figures and Tables

**Table 1:** Ultraselective PSA assays.

Year	Study	Reference	Method	LLD (ng/L)
2023	Ozyurt	[23]	ITO-PET	0.0074
	Wu	[27]	Ag NP	0.001
2022	Cao	[28]	PtNP@Co2O4 NP	10
	Orlov	[29]	Enzyme-linked	19
	Shen	[31]	AIE-ELISA	1.3
2020	Aki	[37]	Membrane biosensor	5
	Cid-Barrio	[38]	Au NP	1
	Farsschi	[40]	Citrate-Ag NPs	70
	Ren	[41]	MesoScale	0.0035
	Wang	[42]	Pd TP	4.2
2019	Hahn	[46]	Au NP	0.1
	Jalalvand	[47]	Aptamer	0.5
	Sun	[50]	Fluorescence	10
	Tian	[51]	Electrochemilumiscence	0.001
2018	Yang	[54]	Sandwich immunosensor	0.003
	Zhou	[55]	Au NP	0.12
2017	Heydari-Bafrooei	[57]	Au NP	1
	Liu	[58]	Au BN sensor	0.27
2015	Kavosi	[70]	Au NP	10
	Liang	[71]	Ag NP	0.004
	Tang	[73]	Au NP	NR
2014	Chen	[76]	Au NP	1
2012	McDermed	[86]	Immuno-PCR	0.27
2011	Wilson	[90]	Digital immunoassay	0.65
2009	Lee,	[95]	Polyclonal Ab	1
	Mani	[96]	Au NP	0.5
	Thaxton	[97]	Au NP	0.33
1996	Ferguson,	[123]	Bayer Immuno 1 PSA	3
1995	Khosravi	[125]	Polyclonal Ab	12
	Mione	[126]	Polyclonal Ab	9.8
	Schambeck	[127]	Immunolite	3.8
1994	Klee	[129]	Monoclonal Ab	8
1993	Arai	[130]	IMx PSA	4
	Iiedtke	[131]	MARKIT-M PSA	200
	Yu	[133]	Tb fluorometry	2
1992	Vessella	[134]	Abbot IMx PSA	30

Abbreviations: Ab—antibody; Ag—silver; Au—gold; ELISA—enzyme-linked immunosorbent immunoassay; ITO-PET—indium tin-oxide polyethylene terephatlate; LLD—lowest limit of detection; NP—nanoparticle; NR—not reported; Pd—palladium; Tb—terbium; TP triangular plates. Abbott IMx—Abbott Laboratories, Abbott Park, IL, USA; Bayer Immuno 1—Business Group, Diagnostics, Bayer Group, Tarrytown, NY, USA.

**Table 2:** Clinical studies.

Year	Study	Reference	Patients				*p* Values for Outcmes		Hazard Ratio
			Total Number	Median Age at Diagnosis (Years)	Outcomes (Numbers)				
					PSAR	Deaths	PSAR	OS	
2023	Bokemann	[20]	1509	74	NR	760	NR	NR	NR
	Hussain	[21]	1409	74	NR	364	NR	NR	NR
	Lee	[22]	397	59	126	NR	<0.00001	NR	NR
	Sutil	[24]	427	64	NR	72	NR	NR	NR
	Tilki	[25]	759	64	NR	127	NR	0.008	1.49
	Ueno	[26]	418	67	51	NR	0.001	NR	NR
2022	Saad	[30]	1401	74	NR	NR	NR	NR	NR
	Von Eyben	[32,33]	1216	68	NR	133	NR	<0.0001	NR
2021	Chung	[34]	1483	64	323	NR	<0.001	NR	NR
	Jansen	[35]	315	70	NR	NR	NR	NR	NR
	Zakaria	[36]	167	61	23	NR	0.013	NR	2.7
2020	Dess	[39]	760	65	238	104	NR	0.004	1.59
2019	Bottke	[43]	301	66	92	17	NR	0.004	3
	Bryant	[44]	764	66	95	25	NR	NR	NR
	Grivas	[45]	213	63	103	NR	<0.01	NR	NR
	Pike	[48]	204	NR	NR	160	NR	<0.001	5.07
	Schroeder	[49]	459	NR	75	24	0.01	0.006	NR
2018	Burkhardt	[52]	102	NR	75	2	NR	NR	NR
	Kang	[53]	269	NR	137	21	<0.0001	<0.001	NR
2017	Abugharib	[56]	657	NR	198	135	<0.0001	0.002	NR
	Royce	[59]	157	72	85	110	NR	<0.01	NR
	Skove	[60]	1790	NR	820	NR	<0.001	NR	NR
	Vessely	[61]	205	NR	106	NR	<0.01	NR	NR
	Von Eyben	[62]	1	50	NR	NR	NR	NR	NR
2016	Boehm	[63]	5619	65	1014	181	NF	NR	NR
	Fossatti	[64]	716	64	129	NR	<0.001	NR	NR
	Laajaala	[65]	503	NR		NR	NR	NR	NR
	Naik	[66]	532	64	354	185	<0.0001	>0.05	NR
	Sokolls	[67]	754	69	262	NR	NR	NR	NR
	Stish	[68]	1106	61	669	183	<0.001	NR	NR
2015	Kang	[69]	247	67	114	NR	<0.0001	NR	NR
	Sikkula	[72]	604	NR	365	NR	<0.01	NR	1.12
	Tilki	[74]	14,532	64	2950	NR	<0.0001	NR	NR
2014	Briganti	[75]	472	NR	126	NR	NR	NR	NR
	Furubayashi	[77]	200	66	17	NR	<0.0001	NR	NR
	Jackson	[78]	409	65	261	49	NR	NR	NR
	Keto	[79]	294	65	NR	31	NR	<0.0001	NR
	Mir	[80]	2348	67	177	31	<0.0001	0.0098	NR
	Vessely	[81]	116	60	55	NR	NR	NR	NR
2013	Vesely	[82]	319	NR	107	31	NR	NR	NR
2012	D’Amico	[83]	734	63	NR	201	NR	NR	NR
	Ho	[84]	1038	69	262	NR	<0.001	NR	NR
	Lepor	[85]	34	60	11	NR	NR	NR	NR
	Siegmann	[87]	301	61	82	3	NR	NR	NR
	Yoshida	[88]	102	66	22	NR	>0.001	NR	NR
2011	Malik	[89]	1197	59	32	NR	0.001	NR	NR
2010	Chang	[91]	115	58	NR	NR	NR	NR	NR
	Eisenberg	[92]	525	65	87	NR	<0.001	NR	NR
	Hong	[93]	384	NR	63	NR	<0.001	NR	NR
	Moreira	[94]	2735	65	635	NR	<0.01	NR	NR
2009	Viney	[98]	300	NR	70	NR	<0.001	NR	NR
	Wiegel	[99]	162	62	75	NR	NR	NR	NR
	Zelefsky	[100]	844	66	NR	65	NR	NR	NR
2007	Kinoshita	[101]	257	68	49	NR	NR	NR	NR
	Shimizu	[102]	257	65	23	NR	NR	NR	NR
	Stephenson	[103]	1540	NR	1047	NR	0.003	NR	NR
2006	Ray	[104]	4839	NR	2318	NR	NR	NR	NR
	Sakai	[105]	177	NR	16	NR	<0.0001	NR	NR
	Stephenson	[106]	3125	NR	458	NR	NR	NR	NR
	Taylor	[107]	225	690	21	NR	NR	NR	NR
2005	Nakamura	[108]	46	NR	15	0	<0.001	NR	NR
	Shen	[109]	545	67	54	NR	<0.05	NR	NR
2003	Shinhal	[110]	14	60	0	NR	NR	NR	NR
2000	Doherty	[111]	134	64	49	NR	<0.001	NR	NR
	Vassilikos	[112]	NR	64	NR	NR	NR	NR	NR
1999	Allard	[113]	384	NR	49	4	<0.0005	NR	NR
	Hase	[114]	442	NR	88	NR	NR	NR	NR
	Pound	[115]	1997	NR	315	NR	NR	NR	NR
1998	Arai	[116]	34	NR	NR	16	NR	NR	NR
	Garg	[117]	78	NR	21	NR	<0.0001	NR	NR
	Morris	[118]	159	NR	2	NR	NR	NR	NR
1997	Ellis	[119]	170	NR	NR	24	NR	NR	NR
	Pruthi	[120]	31	NR	31	NR	NR	NR	NR
	Whiterspoon	[121]	127	NR	66	NR	NR	NR	NR
	Yu	[122]	148	65	51	NR	NR	NR	NR
1996	Van Irrsel	[124]	137	NR	NR	12	NR	NR	NR
1995	Yu	[128]	15	NR	10	NR	NR	NR	NR
1993	Stamey	[132]	187	NR	22	NR	NR	NR	NR
Total number			67,489		14,865	2663			

NR—not reported.

## Data Availability

We have not sent our database to a national archive.

## Supplementary Materials

The following supporting information can be downloaded at: https://www.mdpi.com/article/10.3390/biomedicines12040822/s1, Table S1: All studies. References [20,21,22,23,24,25,26,27,28,29,30,31,32,33,34,35,36,37,38,39,40,41,42,43,44,45,46,47,48,49,50,51,52,53,54,55,56,57,58,59,60,61,62,63,64,65,66,67,68,69,70,71,72,73,74,75,76,77,78,79,80,81,82,83,84,85,86,87,88,89,90,91,92,93,94,95,96,97,98,99,100,101,102,103,104,105,106,107,108,109,110,111,112,113,114,115,116,117,118,119,120,121,122,123,124,125,126,127,128,129,130,131,132,133,134] are mentioned in the Supplementary Materials.
