# Supplementary material for: Prostate-Specific Antigen as an Ultrasensitive Biomarker for Patients with Early Recurrent Prostate Cancer: How Low Shall We Go? A Systematic Review
Source: Biomedicines. 2024 Apr 8;12(4):822. doi: 10.3390/biomedicines12040822 (PMC11048591; doi:10.3390/biomedicines12040822)
Supplement: Supplementary file 1 [file biomedicines-12-00822-s001.zip › biomedicines-2639545-supplementary.pdf]

Supplement **Table S1.** All studies.

Publication			Result	
Year	Author	Reference	Number of patients	Median age at diagnosis
2023	Bogemann	[20]	1509	74
	Hussain	[21]	1409	74
	Lee	[22]	397	63
	Ozyurt	[23]	5	NR
	Sutil	[24]	427	64.5
	Tilki	[25]	759	64
	Ueno	[26]	418	67
	Wu	[27]	NR	NR
2022	Cao	[28]	NR	NR
	Orlov	[29]	NR	NR
	Saad	[30]	1401	74
	Shen	[31]	NR	NR
	Von Eyben	[37, 38]	1216	68
2021	Chung	[34]	1493	64
	Jansen	[35]	315	70
	Zakaria	[36]	167	61
2020	Aki	[37]	NR	NR
	Cid-Barrio	[38]	NR	NR
	Dess	[39]	760	65
	Farschi	[40]	NR	NR
	Ren	[41]	20	61
	wang	[42]	NR	NR
2019	Bottke	[43]	301	66
	Bryant	[44]	764	66
	Grivas	[45]	213	63
	Hahn	[46]	NR	NR
	Jalalvand	[47]	NR	NR
	Pike	[48]	204	NR
	Schroeder	[49]	459	NR
	Sun	[50]	NR	NR
	Tian	[51]	NR	NR
2018	Burkhardt	[52]	102	NR
	Kang	[53]	269	NR
	Yang	[54]	NR	NR

	Zhou	[55]	NR	NR
2017	Abugharib	[56]	657	NR
	Heydari-Bafrooei	[57]	NR	NR
	Liu	[58]	NR	NR
	Royce	[59]	73	73
	Skove	[60]	1939	NR
	Vesely	[61]	295	
	Von Eyben	[62]	1	57
2016	Boehm	[63]	5617	65
	Fossati	[64]	716	NR
	Laajala	[65]	555	64
	Naik	[66]	532	64
	Sokoll	[67]	754	61
	Stish	[68]	1106	67
2015	Kang	[69]	247	NR
	Kavosi	[70]	NR	NR
	Liang	[71]	NR	NR
	Seikkula	[72]	604	64
	Tang	[73]	NR	NR
	Tilki	[74]	14532	64
2014	Briganti	[75]	472	62
	Chen	[76]	NR	NR
	Furubayashi	[77]	200	66
	Jackson	[78]	448	65
	Keto	[79]	294	65
	Mir	[80]	2348	60
	Vesely	[81]	116	NR
2013	Vesely	[82]	319	NR
2012	D'Amico	[83]	734	63
	Ho	[84]	1038	69
	Lepor	[85]	31	61
	McDenmed	[86]	NR	NR
	Siegmann	[87]	301	66
	Yoshida	[88]	102	NR
2011	Malik	[89]	1197	58
	Wilson	[90]	33	64
2010	Chang	[91]	115	MR
	Eisenberg	[92]	525	NR
	Hong	[93]	206	65
	Moreira	[94]	2735	NR
2009	Lee	[95]	NR	NR

	Mani	[96]	NR	NR
	Thaxton	[97]	NR	NR
	Viney	[98]	300	NR
	Wiegel	[99]	162	66
	Zelefsky	[100]	844	66
2007	Kinoshita	[101]	257	65
	Shimizu	[102]	182	NR
	Stephenson	[103]	1540	NR
2006	Ray	[104]	4839	NR
	Sakai	[105]	177	69
	Stephenson	[106]	3125	NR
	Taylor	[107]	225	69
2005	Nakamura	[108]	46	NR
	Shen	[109]	905	60
2003	Shinghal	[110]	15	60
2000	Doherty	[111]	200	61
	Vassilikos	[112]	197	NR
1999	Allard	[113]	155	NR
	Haese	[114]	482	NR
	Pound	[115]	1997	NR
1998	Arai	[116]	34	NR
	Garg	[117]	78	NR
	Morris	[118]	159	NR
1997	Ellis	[119]	170	NR
	Pruhti	[120]	31	NR
	Yu	[122]	148	NR
	Witherspoon	[121]	127	NR
1996	Ferguson	[123]	NR	NR
	Van Irsel	[124]	137	NR
1995	Khosravi	[125]	NR	NR
	Mione	[126]	NR	NR
	Schambeck	[127]	111	NR
	Yu	[128]	15	NR
1994	Klee	[129]	NR	NR
1993	Arai	[130]	NR	NR
	iiedtke	[131]	NR	NR
	Stamey	[132]	187	NR
	yu	[133]	NR	NR
1992	Vessella	[134]	NR	NR
Total number			65489	

Abbreviation: NR = not reported
